# Experimental study on loading combination of filling body and surrounding rock

**DOI:** 10.1038/s41598-025-03927-3

**Published:** 2025-05-28

**Authors:** Dong Li, Jucai Chang, Fenghui Li, Yaoshan Bi, Shunjie Huang, Litong Dou

**Affiliations:** 1 School of Mechanical and Electrical Engineering, Huainan Normal University, Huainan, 232038 China; 2 School of Mining Engineering, Anhui University of Science and Technology, Huainan, 232000 China; 3 School of Civil and Transportation Engineering, Ningbo University of Technology, Ningbo, 315211 China

**Keywords:** Filling body, Combination, Cyclic loading and unloading, Damage variable, Materials science, Engineering, Civil engineering

## Abstract

To investigate the deterioration characteristics of filling body combinations and surrounding rock in excavating technology without coal pillars in the original roadway filling, uniaxial loading destructive tests and cyclic loading and unloading tests were conducted with various combinations of two, three, and four lithologies to analyze strength characteristics, energy storage, and dissipation laws. The results indicate that when the filling is combined with another lithology, strengthening degree of the filling is negatively correlated with the height of other lithology; the higher the strength of the material combined with the filling, the greater the amplitude of the weakening. The filling exhibits a faster speed and higher upper limit of elastic energy storage compared to mudstone. When filling is combined with two or three lithologies, the strength weakening degree is negatively correlated with their height ratio. However, under same cycles, elastic energy of coal-free combinations has a faster storage speed and a higher upper limit, while coal-containing combinations exhibit higher energy dissipation. During tests, the elastic energy of different lithological combinations is linearly related to the number of cycles, and the energy is divided into four stages. The damage variable is exponentially related to the height of the roof and floor. The intrinsic model was modified using the stress-strain relationship considering the internal damage factor in front of the peak and verified to accurately simulate the deformation damage of the combined body.

## Introduction

Coal mining has progressively shifted to deeper regions of the mine over the years. Under the compounded effects of high in-situ stress and intensively dynamic pressure, the stress of surrounding rock of gob-side entry increases, leading to an expansion of the fracture zone. The two conventional roadway protection methods have some problems, such as large deformation of roadway surrounding rock, high convergence rate of section and difficult maintenance, which seriously restrict the efficient mining of coal. Combined with the advantages of two methods, excavating technology without coal pillars in the original roadway filling was proposed to deal with the problem of surrounding rock control of deep roadway. The principle of this technology is as follows: during mining of upper working face, a filling stencil is set along the non-mining side of haulage gateway, and then the quick-setting material mixed by filling station is transported to the stencil through two pipelines to form a filling body wall. After the filling body and overlying rock are stability, the return airway in the lower section is excavated along the edge of the filling, thereby achieving the sequential replacement of the non-pillar sections. The filling body in this technology carries the load together with roadway surrounding rock and is subjected to multiple loads during the movement of overlying strata. Thus, the combined deterioration law of the filling and surrounding rock under multiple loads is highly significant for the stability of roadway surrounding rock.

The research on combined bearing is typically conducted through laboratory mechanical tests of the combined body. Currently, there are numerous experimental studies on assemblage, particularly regarding rock burst and hard roof. Many scholars have undertaken extensive research on coal-rock assemblage, primarily focusing on the combination of sandstone and coal. Zuo and Song et al.^1–3^ developed the pre-peak and post-peak crack evolution models of the combination, as well as the differential energy instability analysis model for the coal-rock system through experiments. Their work has significantly contributed to understanding the deformation and damage characteristics and evaluating the tendency for coal rock bursts. Based on two lithologic assemblages of coal and rock, Yu et al.^4,5^ examined the mechanical behavior and fracture evolution of the “rock-coal-rock” and “coal-rock-anchor” combinations formed by sandstone and coal under uniaxial tests. Chen and Yang et al.^6,7^ explored the energy evolution and acoustic emission characteristics of coal-rock combinations with varying height ratios in uniaxial tests. Gong et al.^8^ conducted low loading rate tests on coal-rock combinations to uncover the mechanisms behind burst tendencies. Chen et al.^9^ constructed a mechanical model for two and three lithology combinations formed by hard rock and coal, analyzing the energy accumulation patterns of different parts before failure. Wang et al.^10^ investigated the mechanical parameters of rock-filling assemblies under uniaxial conditions and performed numerical simulations to study the characteristics of microcrack evolution.

Both the filling and the coal are influenced by multiple transfers of overburden loads, and many researchers typically employ cyclic loading and unloading tests to investigate them. Regarding single lithology, Wu et al.^11^ performed an analysis of the mechanical characteristics and energy evolution laws of granite under various graded cyclic loading and unloading conditions. Chen et al.^12^ examined the energy evolution of gas-containing coal under different cyclic loading and unloading stress paths. Jiang et al.^13^ conducted fatigue tests on salt rock under cyclic loading and unloading rates and established a rate effect equation. Scholars et al.^14–19^ investigated the fracture development and failure characteristics of siltstone, jointed sandstone, water-bearing sandstone, sandy mudstone, argillaceous quartz siltstone, and granite during cyclic loading and unloading. Zhao et al.^20^ assessed the energy of sandstone with varying height-diameter ratios and proposed a new method. Concerning multi-lithology assemblages, Xu et al.^21^ explored the failure mechanism of coal-rock assemblages from an energy perspective, based on cyclic loading and unloading tests. Peng and Gao et al.^22^ also analyzed the failure characteristics and fracture development of coal-rock combinations with different coal-rock ratios under uniaxial compression using L-shaped grinding tools. Guo et al.^23^ conducted cyclic impact tests on concrete-surrounding rock combinations to analyze the changes in energy and damage characteristics. Zhao et al.^24^ performed uniaxial mechanical tests on filling material combinations with varying ratios of lime and sand through acoustic emission to investigate their coordinated deformation and activity characteristics. Jiang et al.^25^ analyzed the development of hydraulic fractures and the acoustic emission characteristics of the assembly, considering the interface effect. Wang et al.^26^ analyzed the characteristics of temperature changes in infrared radiation and the alterations in infrared thermal imaging of the combination of the filling body and surrounding rock during cyclic loading and unloading, successfully predicting the development of fractures and failure in the combination. The study investigates the mechanical behavior of rock mass under seepage environment conditions, specifically focusing on the excavation and unloading processes. By conducting triaxial cyclic loading failure tests on rock samples subjected to cyclic pore water pressure, the research aims to evaluate how varying pore pressure levels and unloading rates influence the deformation characteristics and energy evolution of the rock samples under dynamic cyclic loading conditions^27^. This study investigates the impact of cyclic loading on the mechanical properties of damaged coal and the evolution of flaw structures, such as pores and cracks, within the coal. A micro-fracture size judgment index was defined based on fracture volume to determine the dominant macroscopic failure mode in coal bodies^28^. Niu et al.^29^ were carried out triaxial cyclic loading tests on sandstone and granite at different strain amplitudes to investigate the variation of rock internal energy. It can be seen that different scholars for a single lithology material (such as granite, etc.) is mainly combined with other influencing factors, for a variety of lithology is now also gradually increase the experimental influencing factors to analyze the impact of the results of cyclic loading and unloading.

The aforementioned research primarily focused on the cyclic loading and unloading tests of various single lithologies, uniaxial loading tests, and cyclic loading and unloading tests of coal-rock combinations. However, concerning the joint bearing of the filling and surrounding rock in the technology of excavating without coal pillars in the original roadway filling, the combined deformation and damage characteristics of its combinations differed from those of coal-rock combinations. This aspect remains under-explored in the literature. This article examines the various combinations of lithologies and filling as the research subject and investigates their deformation and failure characteristics under cyclic loading and unloading.

## Sample preparation and scheme design

In similar simulation experiments of water-bearing strata, water bags, fluid-solid coupling materials, or independent spring groups are generally used^14–16^. In this experiment, a drainage module combining porous materials and water bags was fabricated. The principle of a connector was used to regulate the water pressure of the drainage module. Based on existing research^17–20^, acoustic emission monitoring and surface displacement observation were employed throughout the experimental process.

### Engineering model of the test combinations

**Fig. 1:**
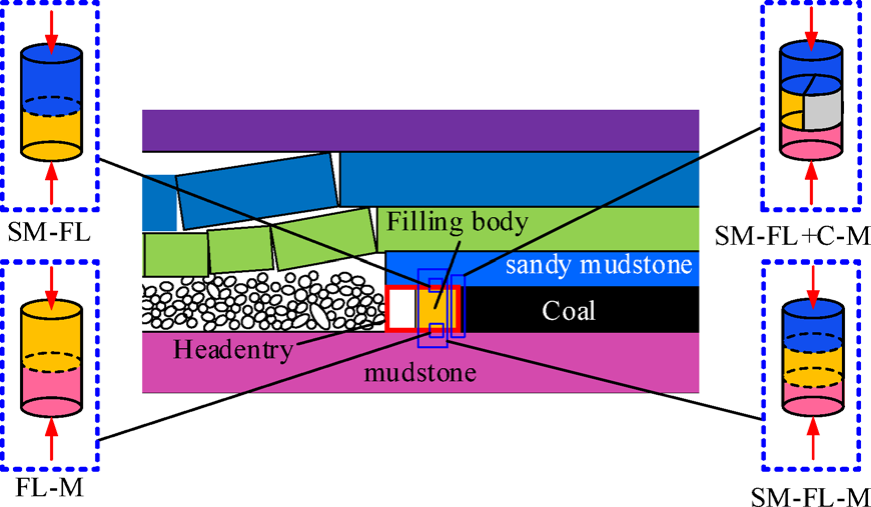
Combined body engineering model.

In the original roadway filling without coal pillars excavation, the primary process of protection roadway technology involves arranging the filling body along the non-mining side of the headentry when the upper working face is mined. Once the mining of the working face is completed and the overburden stabilizes, the return airway in the lower working face is excavated along the edge of the filling, thereby achieving sequential replacement of pillar-free sections. Before and after excavation, various combinations exist between the roof, floor, and filling, as illustrated in Fig. 1.

Compared to gob-side entry, this method differs from the traditional coal pillar protection technique for roadways. Unlike gob-side entry retained, it avoids the issue of subsequent repair of the headentry for repeating disturbance. In the technique, both the filling and surrounding rock share the load before and after the excavation of the new return airway. During mining activities, these are subjected to multiple stress transfer loads from overburden rocks, particularly in four combinations: “sandy mudstone-filling “, “filling-mudstone”, “sandy mudstone-filling -mudstone” and “sandy mudstone-filling + coal-mudstone”. However, the last combination does not exist after new return airway excavation. Consequently, this paper primarily focus on the experimental investigation of these four combinations under cyclic loading and unloading conditions.

### Sample preparation

Given the considerable number of specimens of varying lithologies required for the tests, the rock materials employed here are rock-like materials equipped with orthogonal tests^30^, in which the filling materials have been developed by China University of Mining and Technology and are divided into material A and material B.

The production of standard specimens requires electronic scales, beakers, standard specimen molds, vibration table and other tools. Mix the aggregate and binder together and stir well, add water according to the ratio and mix well, then pour into the mill, placed on the vibration table and stir for 30 s to minimize the air bubbles in the mixture. Interfacial bonding between any two lithologies is achieved by self-bonding of the rock layers. When the lower layer is close to solidification (in a paste-like state), the prepared mud is applied to the upper layer to start the consolidation between these lithologies. If not in a pasty state, the chemical reaction of water absorption at the interface would result in an uneven surface, thus causing potential demolding failures. Covered with cling film and left for 24 h, the standard specimens were allowed to form and then demolded, the samples were wrapped in plastic film, and the specimens were placed in a curing box for 28 d. The temperature was set at 20 °C and the humidity at 65%. The samples were then taken out and further processed according to the Standard for Testing Methods for Engineering Rocks (GB/T50266-99), which included grinding operations, with the aim of ensuring that the overall size of each sample body was Φ50 mm × H100 mm.

### Scheme design

In this paper, sandy mudstone is designated as SM, mudstone is designated as M, filling body is designated as FL, and coal is designated as C. Therefore, SM-FL denotes the combination “sandy mudstone-filling,” FL-M denotes the combination “filling-mudstone”. The combination “sandy mudstone-filling-mudstone” is denoted by SM-FL-M. The combination “sandy mudstone-filling + coal-mudstone” is denoted by SM-FL + C-M.

To facilitate a comparison of the mechanical properties exhibited by the cyclic loading and unloading test and the uniaxial test of the combination, it is necessary to conduct three sets of tests. These comprise the uniaxial test of a single lithology, the uniaxial test of the combination, and the cyclic loading and unloading test of the combination.

**Fig. 2:**
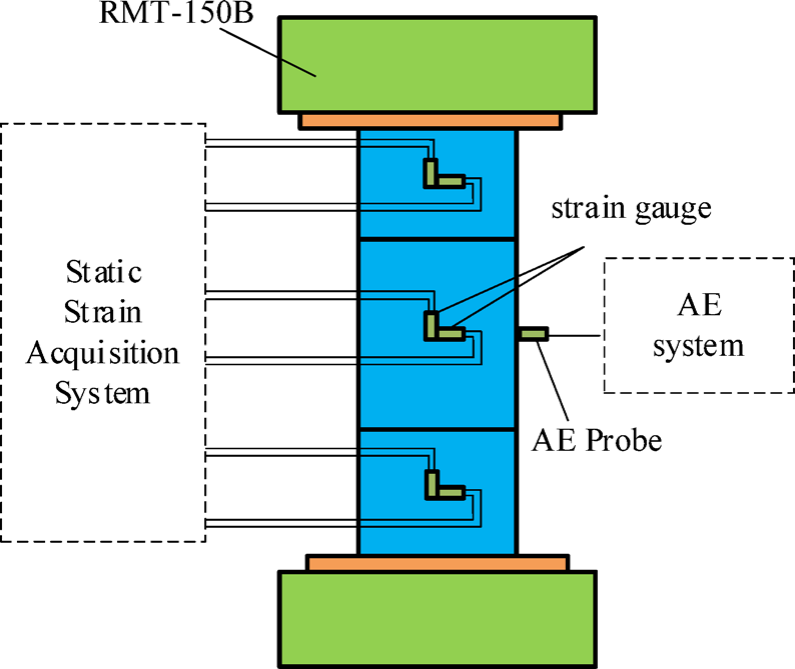
Test system.

The tests were conducted using a rock mechanics testing system (RMT-150 C), and all were controlled by force. An acoustic emission monitoring system was integrated into the test process, in which the acoustic emission system utilized DS5(V2017.06.04a) software, the monitoring system employed eight channels, and the amplifier threshold between the probe and the demodulator was set at 40 dB. In order to better measure the longitudinal deformation and transverse deformation of different lithologies of the specimen, strain gauges were applied to the specimen using AB adhesive, and transverse and longitudinal strain gauges were applied to the center of each lithology layer, as shown in Fig. 2.

Given that the filling body possesses residual strength to support the roof in the field, the lower limit for the cyclic loading was established at 6 kN. The cyclic loading/unloading test of samples was conducted in three steps:

Step 1: The sample should be placed into the test system and applied the first cyclic loading with an upper limit of 7 kN. The load should be applied at a loading rate of 0.05 kN/s.

Step 2: The axial load on the sample should then be unloaded to 6 kN at an unloading rate of 0.05 kN/s, thus completing a complete cycle of loading and unloading.

Step 3: The upper cyclic load limit should be increased by 1 kN per cycle in each subsequent cycle, with the load applied at the same rate and the specimen unloaded at the same unloading rate. This process should be repeated until the sample is damaged.

**Fig. 3:**
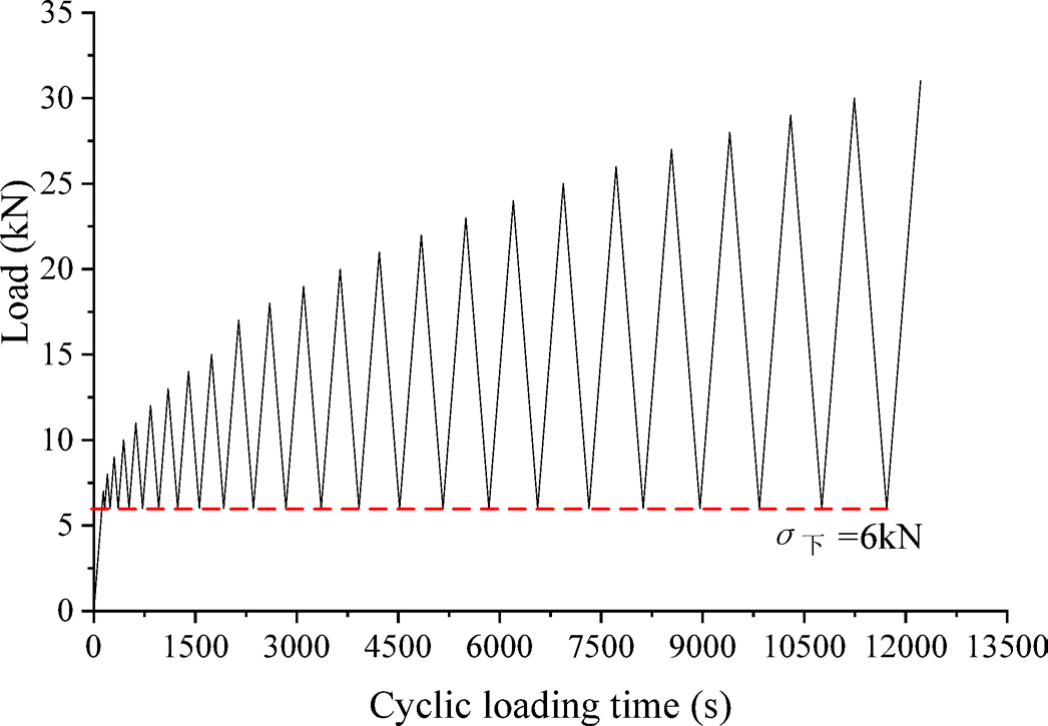
Cyclic loading and unloading paths.

In order to make the whole process of loading and unloading look clearer, the cyclic load loading path is plotted as shown in Fig. 3 below. The specific cyclic loading and unloading scheme is shown in Table 1.

**Table 1:** Test scheme for cyclic loading and unloading of combination samples.

Combination type	Lithology	Height ratio	Lower cycle load(kN)	Loading and unloading rate(kN/s)	Maximum additional load per cycle (kN)
SM-FL	SM, FL	2:3, 1:1, 3:2	6	0.05	1
FL-M	FL, M	2:3, 1:1, 3:2	6	0.05	1
SM-FL-M	SM, FL, M	1:2:2, 3:4:3, 2:2:1	6	0.05	1
SM-FL + C-M	SM, FL, C, M	1:1:1:2, 3:2:2:2, 2:1:1:1	6	0.05	1

## Uniaxial compression test results

### Analysis of results from single lithology samples

According to the results obtained in literature^30^, the single lithologic sample includes the pore compaction stage (if vibrated until there are no bubbles, this stage is not obvious and the gradient is basically close to the second stage), the elastic stage, the elastic-plastic transition stage, the plastic stage, and the failure stage. The average uniaxial compressive strength (UCS) of mix number 4 − 1 is 22.56 MPa, the average elastic modulus is 4.17 GPa, and the tensile strength is 1.54 MPa. The average uniaxial compressive strength (UCS) of mix number 4 − 2 is 15.62 MPa, the average modulus of elasticity is 3.09 GPa, and the tensile strength is 1.62 MPa. These can meet the parameter requirements for preparing combination of sandy mudstone and mudstone respectively.

### Analysis of results from combination samples

Based on the UCS results of the combination, in order to compare the relationship between the average strength peak value of the same combination and the height ratio, and to analyze the dispersion of experimental data, a double Y-axis chart was drawn, as shown in Fig. 3. The UCS of three samples in each group is a bar chart, with red dots representing the average compressive strength of the group and error bars plotted in the chart.

**Fig. 4:**
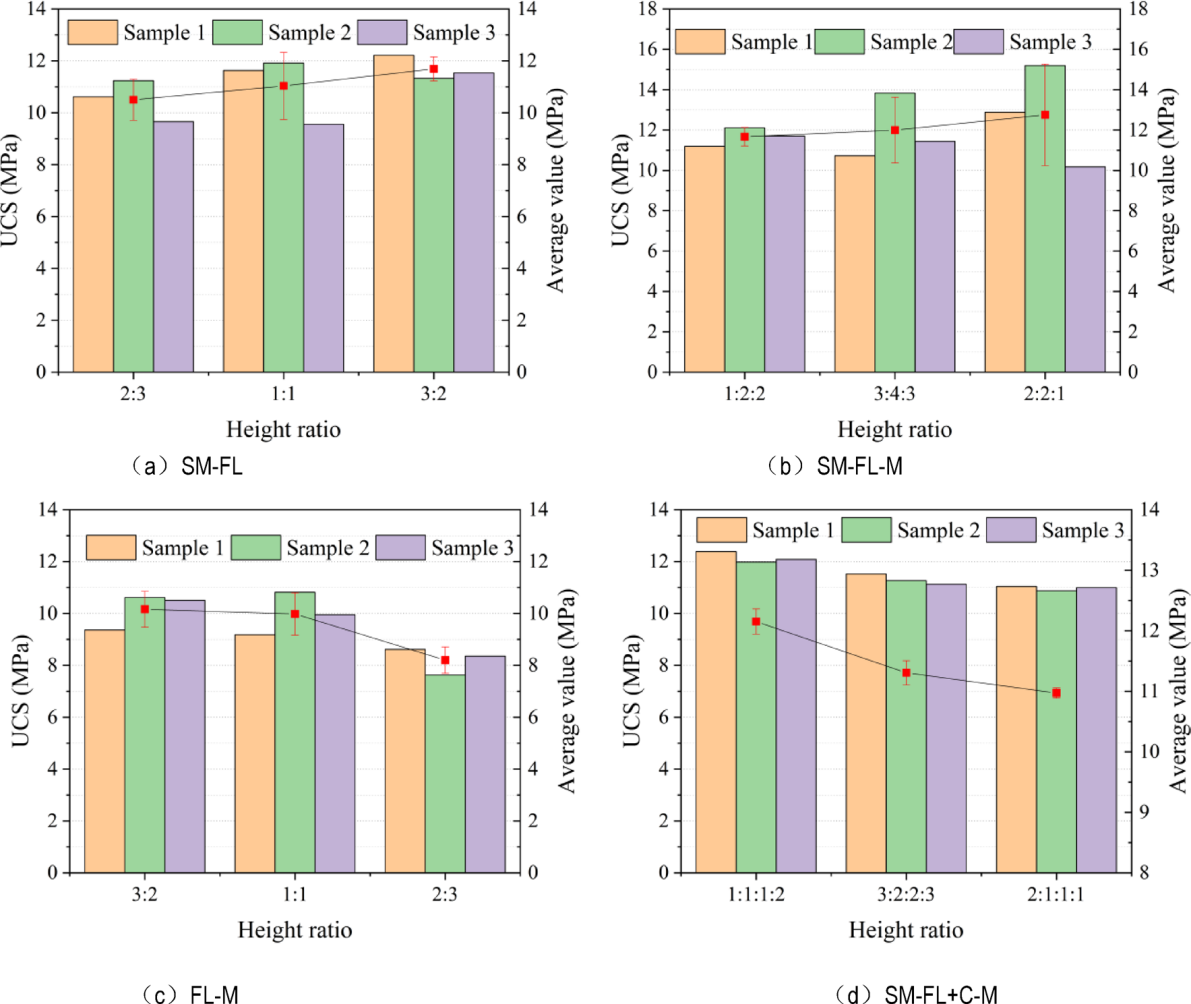
Uniaxial compressive strength and accuracy of combinations.

In Fig. 4a, the average strengths of the SM-FL with height ratios of 2:3, 1:1, and 3:2 are 10.50, 11.03, and 11.69 MPa, respectively, with dispersion coefficients of 7.57%, 11.68%, and 3.96%; it is evident that the strength of the filling body has been enhanced by 61.3%, 63.2%, and 63.8%, respectively, increasing as the proportion of filling body height decreases. Conversely, the strength of sandy mudstone is reduced by 60.8%, 56.5%, and 52.2%, respectively, decreasing as the proportion of sandy mudstone height increases.

In Fig. 4b, the average strengths of the SM-FL-M with height ratios of 1:2:2, 3:4:3, and 2:2:1 are 11.67, 11.997, and 12.753 MPa, respectively. The strength of the filling is enhanced by 63.5%, 68.1%, and 78.7%, while the strength of the mudstone is weakened by 37.1%, 40.5%, and 45.6%, and the strength of the sandy mudstone is weakened by 65.5%, 58.8%, and 52.4%, respectively. It can be observed that while the proportion of the filling remains constant, its strengthening effect is positively correlated with the height ratio of sandy mudstone and mudstone, whereas the degree of weakening of sandy mudstone and mudstone is negatively correlated with the height ratio of sandy mudstone and mudstone.

In Fig. 4c, the average strengths of the FL-M with height ratios of 3:2, 1:1, and 2:3 are 8.20, 10.16, and 9.98 MPa, respectively, with dispersion coefficients of 6.24%, 6.83%, and 8.22%. The strength of the filling is enhanced by 25.9%, 47.6%, and 42.4%, while the strength of the mudstone is weakened by 55.8%, 43.2%, and 39.9%, respectively, decreasing as the proportion of mudstone height increases.

In Fig. 4d, the average strengths of the SM-FL + C-M are 10.972, 11.305, and 12.152 MPa, accompanied by dispersion coefficients of 6.24%, 6.83%, and 8.22%, respectively. The strength of sandy mudstone is diminished by 67.5%, 61.2%, and 54.6%, while the strength of mudstone experiences reductions of 40.8%, 44.0%, and 48.1%, correspondingly. Notably, the degree of weakening in both sandy mudstone and mudstone exhibits a negative correlation with the height ratio between sandy mudstone and mudstone.

The results indicated a positive correlation between the average UCS of SM-FL and FL-M and the height share of the filling body. Additionally, the strength of sandy mudstone was observed to be more susceptible to deterioration than that of mudstone. Furthermore, the average UCS of SM-FL-M demonstrated a positive correlation with the height ratios of sandy mudstone and mudstone (1:2, 1:1, 2:1), and the weakening amplitude of sandy mudstone strength is higher than that of mudstone. Additionally, the average UCS of SM-FL + C-M demonstrated a negative correlation with the height ratio of sandy mudstone and mudstone.

## Results of cyclic loading and unloading tests of combinations

The area enclosed by the loading curve and the lower limit of the cycle represents the input energy (*U*) of the press to the samples. The area enclosed by the unloading curve and the lower limit of the cycle represents the elastic energy (*U*_*e*_) produced by the samples in the cycle. The difference between these two values represents the dissipation energy (*U*_*d*_) produced by the cycle and is utilized for statistical analysis of the characteristics of the different combinations in terms of energy storage and dissipation in the test results of samples.

### Analysis of FL-M test results

**Fig. 5:**
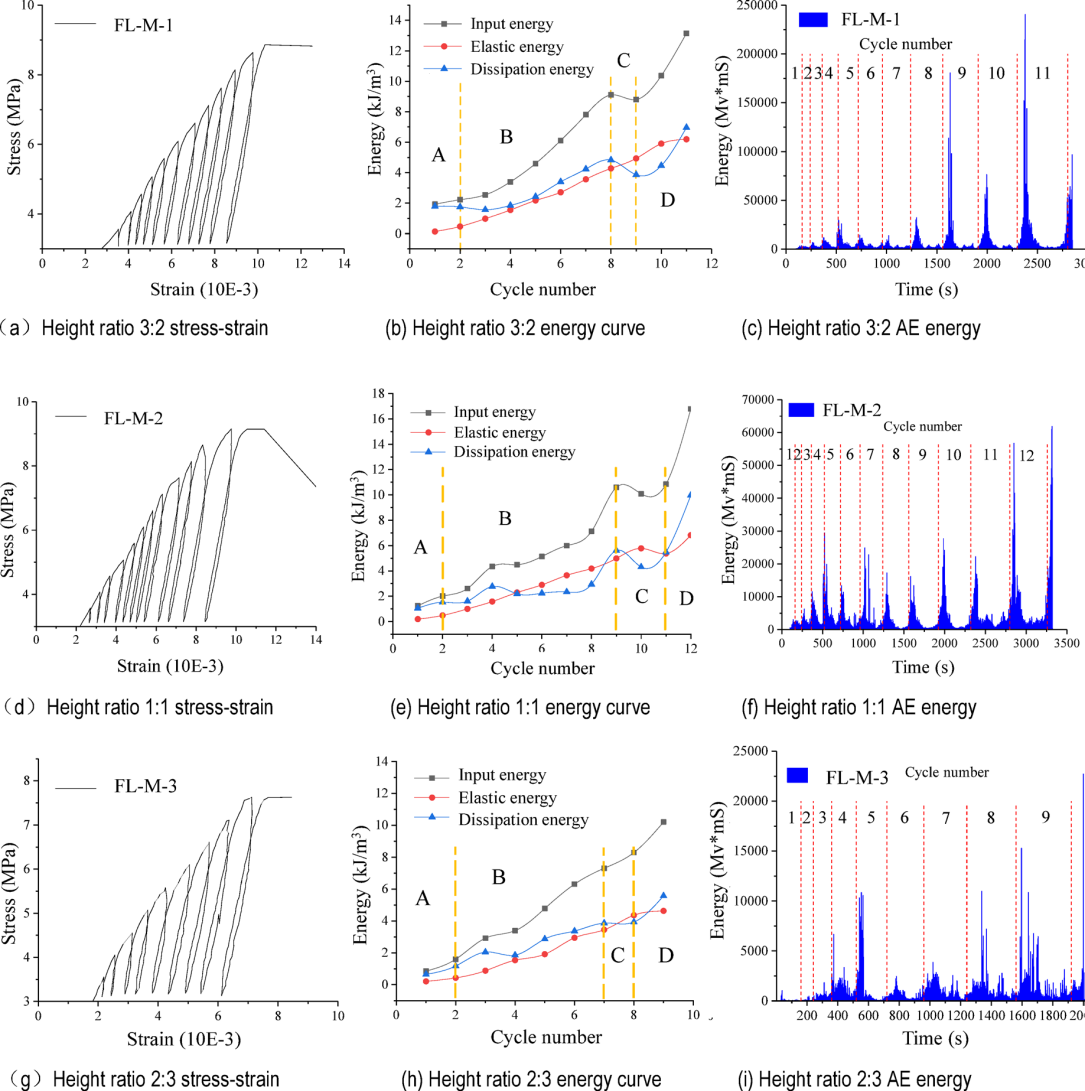
Cyclic loading and unloading stress-strain curves and energy variation of FL-M.

As illustrated in Fig. 5, the stress-strain behavior and energy variations of FL-M with differing height ratios are presented in the test results. Three groups of FL-M samples exhibited damage after completing 11, 12, and 9 full cycles of loading and unloading, respectively. While the overall trend indicates an increase in both input and dissipated energies throughout the cyclic process, these energies can be categorized into four distinct stages: initial energy accumulation (stage A), rapid energy accumulation (stage B), steady energy input (stage C), and rapid energy dissipation (stage D). Each stage exhibits unique characteristics during the entire cycle process.

Stage A: Cycles 1 to 2 of the cyclic loading and unloading test, which constituted approximately 16–22% of the total cyclic process, involved the compaction of the pores within samples, corresponding to the elastic stage of uniaxial compression tests. At the conclusion of the test, the input energy absorbed by samples with height ratios of the filling body to mudstone of 3:2, 1:1, and 2:3 was 4.277 kJ/m³, 4.983 kJ/m³, and 7.302 kJ/m³, respectively. The stored elastic energy was 4.825 kJ/m³, 4.983 kJ/m³, and 3.444 kJ/m³, respectively. It can be seen that the input energy absorbed by samples at the time of damage is negatively correlated with the height ratios of the filling body and mudstone. The stored elastic energy is observed to be at its maximum at the height ratio of 1:1, indicating that the filling absorbs a greater amount of energy than the mudstone. It can be inferred that an increase in the height difference between the filling and mudstone will result in enhanced energy dissipation within the samples.

Stage B: Compared to stage A, the input energy and dissipated energy increase in amplitude and range, accounting for about 55–58% of the entire cycle. In this stage, the elastic energy rises linearly with the increase in input energy, while the dissipated energy grows with the number of cycles.

Stage C: In this stage, the input and dissipated energies show a trend toward stability or decline, while the combination continues to accumulate elastic energy under sustained compression. Simultaneously, there is a notable rise in AE signals within the combination, corresponding to the elastic-plastic transition phase during uniaxial compression. This stage occurs in the final 2 to 3 cycles of the complete process, representing approximately 8–11% of the entire cyclic sequence.

Stage D: This stage encompasses the final 1–2 cycles of the complete cycle preceding damage, accounting for about 11–18% of the entire cyclic process. During this stage, the cyclic load approaches the peak load associated with damage. However, the combination continues to absorb input energy to accumulate energy. Compared to the preceding stages, the density of the cyclic curve decreases, the rate of increase in dissipated energy intensifies significantly, dense fractures appear, and the deformation becomes irrecoverable. Concurrently, the acoustic emission signal reaches its maximum value throughout the entire complete cycle stage. This phenomenon indicates that the combination is on the brink of damage, with internal cracks progressively interconnecting, corresponding to the plastic deformation of the sample in uniaxial compression.

The elastic energy is approximately linearly related to the number of cycles during the initial nine cycles of the entire process, from the start of cycling to the damage of the combination. By means of linear fitting, the following relationship is obtained:

1 $$\left\{ \begin{gathered} FL - M - 1:y=0.643x - 0.868,{R^2}=0.99147 \hfill \\ FL - M - 2:y=0.608x - 0.679,{R^2}=0.98565 \hfill \\ FL - M - 3:y=0.601x - 0.746,{R^2}=0.98245 \hfill \\ \end{gathered} \right.$$

It can be observed that an increase in the height ratio of the filling body leads to a heightened sensitivity of energy changes to variations in the number of cycles. The filling body demonstrates a higher elastic energy storage rate than the mudstone, and based on the stored energy value, it exhibits a superior upper storage limit compared to the mudstone.

### Analysis of SM-FL-M test results

**Fig. 6:**
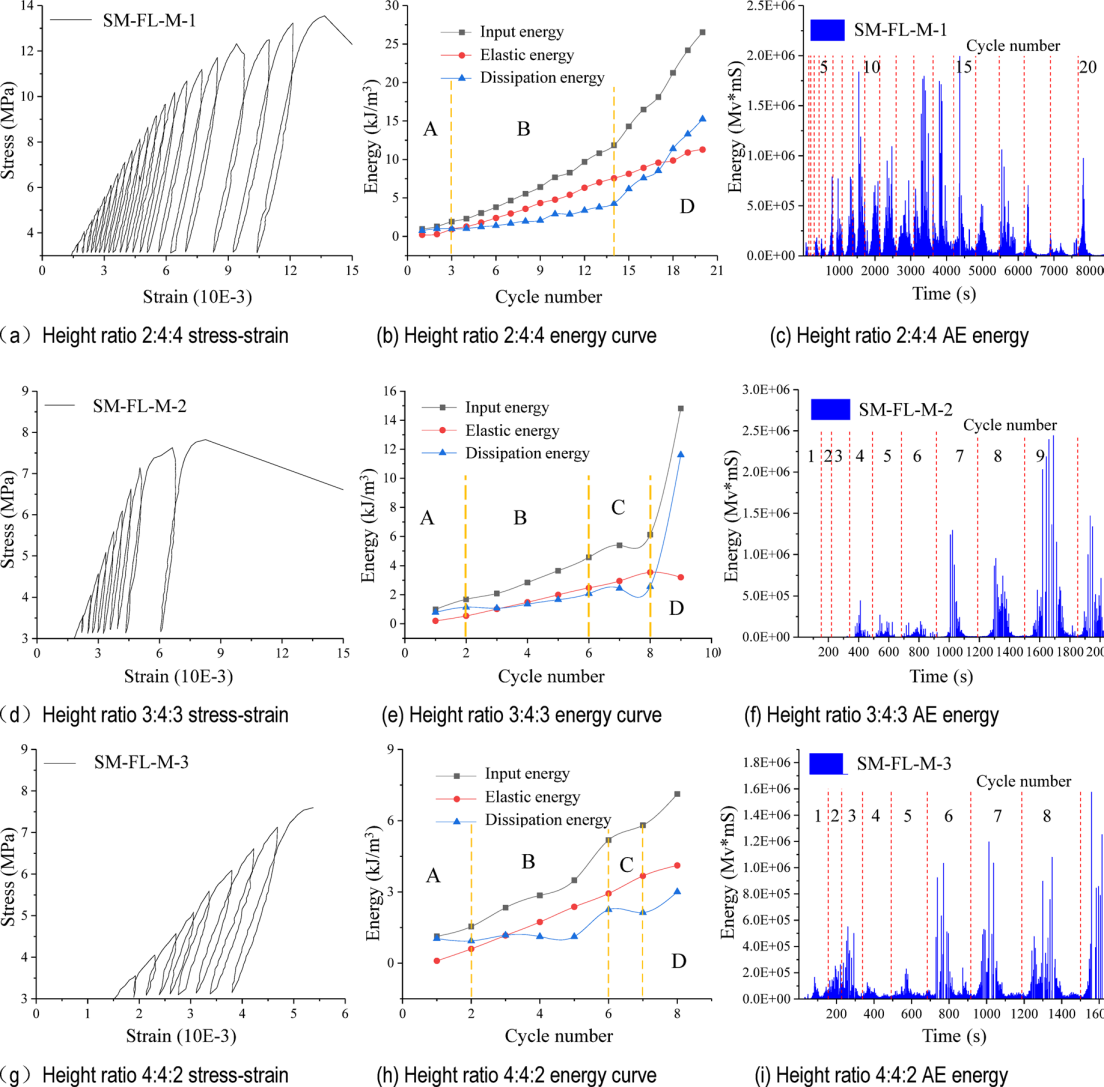
Cyclic loading and unloading stress-strain curves and energy variation of SM-FL-M.

As illustrated in Fig. 6, the stress-strain and energy results of SM-FL-M with varying height ratios are presented in the test results. Similarly, SM-FL-M can be divided into four stages of energy accumulation and dissipation. However, in the absence of a vertical fracture during cyclic loading, unloading does not exacerbate damage to the combination. Instead, it leads to increased energy accumulation and strengthens the sample. Stage C is absent, and there are only three stages: initial energy accumulation, rapid energy accumulation, and rapid energy dissipation.

Like FL-M, the relationship between elastic energy and the number of cycles can be described as approximately linear throughout the entire process, from the start of cycling to the destruction of SM-FL-M. Thus, by linear fitting, the following relationship can be established:

2 $$\left\{ \begin{gathered} SM - FL - M - 1:y=0.615x - 1.088,{R^2}=0.99571 \hfill \\ SM - FL - M - 2:y=0.431x - 0.219,{R^2}=0.96852 \hfill \\ SM - FL - M - 3:y=0.588x - 0.561,{R^2}=0.9982 \hfill \\ \end{gathered} \right.$$

It can be observed that when the height ratio of the filling body is 40%, an increase in the height difference between sandy mudstone and mudstone results in a higher sensitivity of the elastic energy to changes in the number of cycles. At the end of the cycle, a larger proportion of mudstone indicates a higher dissipation energy, suggesting that mudstone is more susceptible to damage than sandy mudstone.

The strengths of SM-FL-M in cyclic loading and unloading were 13.547 MPa, 7.823 MPa, and 7.599 MPa, respectively, for all filling bodies with a height ratio of 40% and varying height ratios of sandy mudstone to mudstone of 1:2, 1:1, and 2:1. The SM-FL-M-1 sample, with a height ratio of 1:2 between sandy mudstone and mudstone, showed no cracks during cyclic loading. Furthermore, its compressive strength improved by approximately 2 MPa compared to UCS, representing an 18% increase. The compressive strengths of SM-FL-M-2 and SM-FL-M-3, with height ratios of 1:1 and 2:1, were reduced due to the formation of vertical fractures in the sandy mudstone region during cyclic loading, which exacerbated the deterioration of the samples during cyclic unloading, leading to a decrease in their strengths.

Comparing the elastic energy under the same cycle, it is evident that the elastic energy stored by combinations increases with the height ratio of sandy mudstone to mudstone (1:2, 1:1, 2:1), with the increase in elastic energy under the same cycle ranging from 10 to 25%. This indicates that the upper limit of elastic energy that can be stored by the combination rises with the height ratio of sandy mudstone. In comparison to mudstone, sandy mudstone can store more elastic energy by absorbing mechanical energy at a given input energy.

### Analysis of SM-FL + C-M test results

**Fig. 7:**
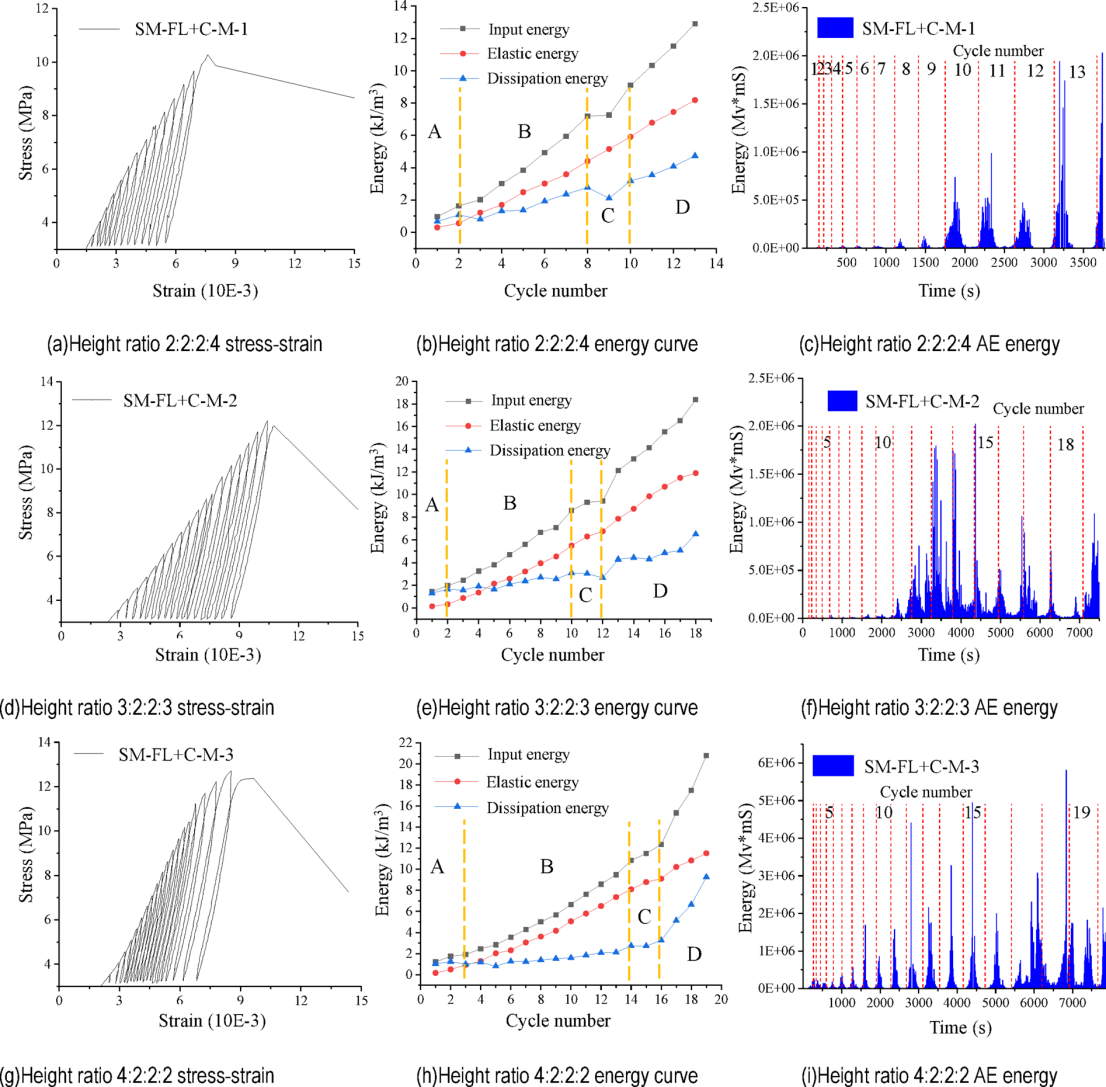
Cyclic loading and unloading stress-strain curves and energy variation of SM-FL-M.

According to the test results, the stress-strain and energy results of SM-FL + C-M with different height ratios are shown in Fig. 7.

The filling and coal volume ratios of SM-FL + C-M are both 20%, while the height ratios of sandy mudstone and mudstone differ, being 1:2, 1:1, and 2:1, respectively. The UCS under cyclic loading and unloading tests are 10.278, 12.722, and 12.213 MPa, respectively. When the height ratio is 1:2, cracks develop in the composite during cyclic loading. Compared to the uniaxial strength, the strength is reduced to some extent, with a decrease of about 6.3%. However, when the height ratio is 1:1, no vertical cracks occur during cyclic loading, and the strength is enhanced, with an increase of about 12.53%. The height ratio of 2:1, similar to SM-FL-M, exhibits vertical and transverse cracks intersecting in sandy mudstone during cyclic loading, worsening the failure of the multi-component combination during cyclic unloading, although the failure strength remains slightly greater than the uniaxial strength.

From the stress-strain curves of cyclic loading and unloading, it is evident that the SM-FL + C-M with a height ratio of 1:2 between sandy mudstone and mudstone is dense in the first five cyclic loading and unloading cycles, with the input energy during the cycle primarily converted into elastic properties, resulting in relatively low dissipated energy. Between cycles 5 and 13, compared to the earlier cycles, there is a slight sparsity, with transverse and inclined cracks continuously appearing on the surface of sandy mudstone. Mudstone exhibits inclined cracks that generate strong AE signals. The height ratio of sandy mudstone to mudstone at 2:1 is denser in the first 16 cycles of the loading and unloading curves, becoming sparser in cycles 16–18 compared to the previous cycles. The sparsity of the 1:1 ratio curve between sandy mudstone and mudstone is not pronounced.

The elastic energy is approximately linearly related to the number of cycles throughout the cycling start to destruction of the combination, similarly to FL-M and SM-FL-M, with the following relationship by linear fitting:

3 $$\left\{ \begin{gathered} SM - FL+C - M - 1:y=0.678x - 0.842,{R^2}=0.99366 \hfill \\ SM - FL+C - M - 2:y=0.732x - 1.508,{R^2}=0.98895 \hfill \\ SM - FL+C - M - 3:y=0.657x - 1.231,{R^2}=0.99242 \hfill \\ \end{gathered} \right.$$

As for sandy SM-FL-M and FL-M, the energy can be divided into four stages. Comparing the elastic energy under the same cycles, it can be found that the stored elastic energy decreases with the increase of sandy mudstone to mudstone height ratio (1:2, 1:1, 2:1) in SM-FL + C-M, and the decrease of elastic energy is in the range of 4–54% under the same cycles, which indicates the increase of dissipated energy due to the damage and deformation of the coal. The two laws are opposite in SM-FL + C-M compared to SM-FL-M. The stored elastic energy decreases with increasing height ratio of sandy mudstone.

## Damage analysis

From the above analysis of the stress-strain relationship of the specimens, it can be seen that the specimen, under the action of pressure, first experiences the compaction of bubbles and fissures in the interior, and then produces cracks after the elastic phase, and then macro-destruction, from which damage occurs in the process. According to the definition of the damage factor of dissipated energy method, the definition of the damage factor of dissipated energy method is the ratio of the dissipated energy of the m-time of the cycle to the cumulative dissipated energy, the damage factor of the m-time of the cycle is ${D_i}=U_{m}^{d}/U_{i}^{d}$, and the cumulative damage factor is $D=\sum\nolimits_{1}^{m} {{D_i}}$ .

**Fig. 8:**
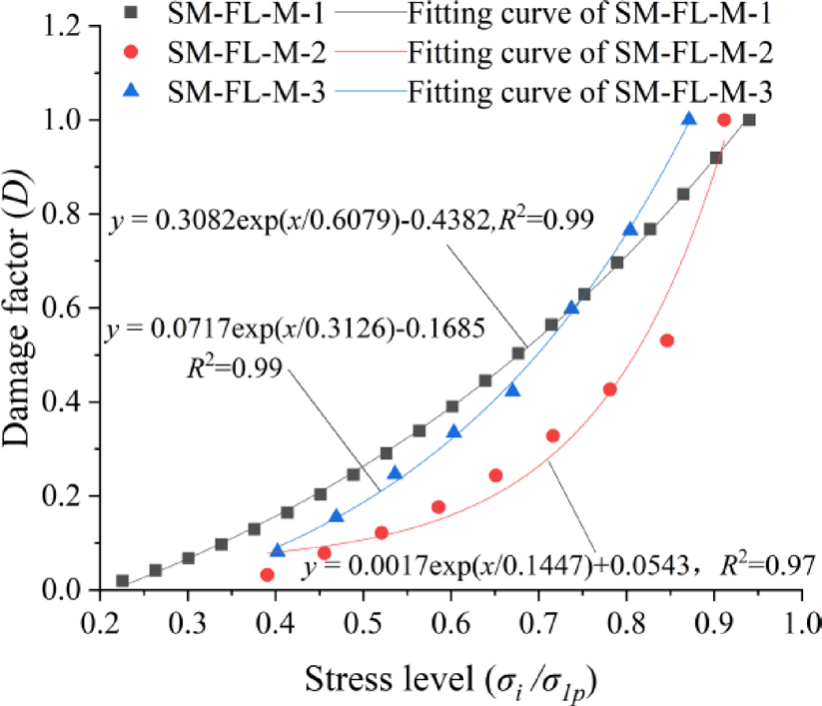
The damage factor of SM-FL-M *D* vs. stress level.

According to the formula of the dissipated energy method and combined with the previous calculation results of the dissipated energy, we can obtain the relationship curve between the damage factor and the stress level, as shown in Fig. 8.

The damage factor and stress level can be fitted to the combination of the three lithologies for the three height ratios:

4 $$D = 0.0851\exp \left( {\frac{{\sigma _{i} /\sigma _{{1p}} }}{{0.3558}}} \right) - 0.14511,\,R^{2} = 0.9166$$

where σ_1p_ is the peak stress and σ_i_ is the arbitrary moment stress.

A numerical linear fit of the peak stress and the ratio of sandy mudstone h_SM_ to mudstone height h_M_ yields the following equation:

5 $${\sigma _{1p}}=0.72686{h_{SM}}/{h_M}+11.292,{R^2}=0.9976$$

By incorporating it into the damage factor, the numerical relationship between the damage factor D and the axial stress σ_i_ can be obtained:

6 $$D=0.0851\exp \left( {\frac{{{\sigma _i}{h_M}}}{{0.2586{h_{SM}}+4.0177{h_M}}}} \right) - 0.14511$$

**Fig. 9:**
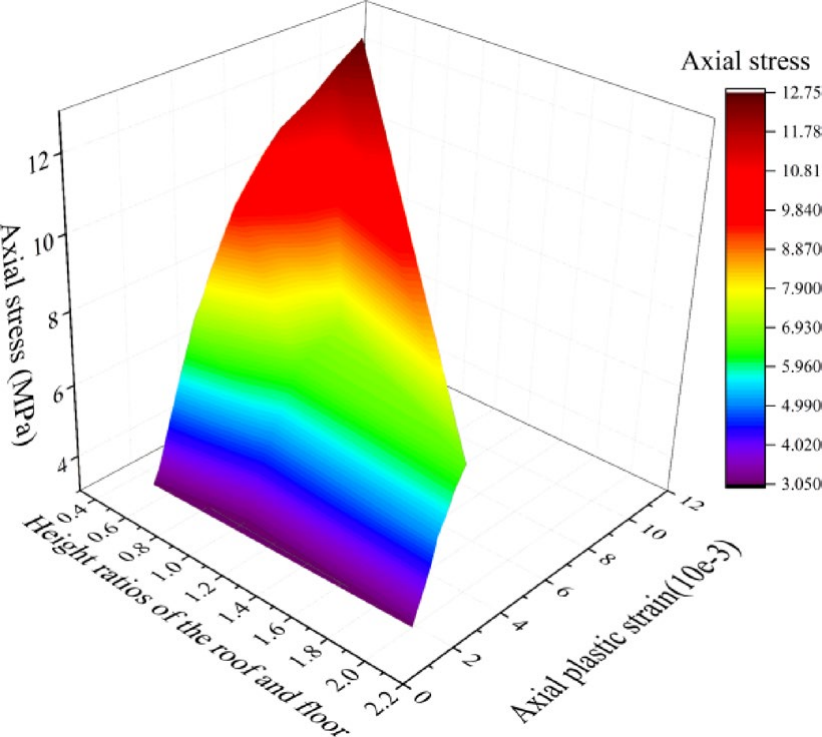
Relationship between axial stress, plastic strain and the height ratio of the roof and floor.

For the surface fitting of axial stress, height ratio of sandy mudstone and mudstone, and plastic strain, as shown in Fig. 9, the specific numerical expression is as follows:

7 $${\sigma _i}= - 95.3253+235.7156\exp \left( { - \frac{1}{2} \times {{\left( {\frac{{{h_{SM}}+289.2492{h_M}}}{{231.5867{h_M}}}} \right)}^2} - \frac{1}{2} \times {{\left( {\frac{{{{\overline {\varepsilon } }^p} - 10.3241}}{{20.40134}}} \right)}^2}} \right)$$

Therefore, we can obtain the relationship between damage factor and plastic strain, which is expressed as follows:

8 $$D=0.0851\exp \left[ {\left( { - 95.3253+235.7156 \times \frac{1}{{\exp \left( {{{\left( {\frac{{{h_{SM}}+289.2492{h_M}}}{{926.3468{h_M}}}} \right)}^2}+{{\left( {\frac{{{{\overline {\varepsilon } }^p} - 10.3241}}{{81.6052}}} \right)}^2}} \right)}}} \right)\left( {\frac{{{h_M}}}{{0.2586{h_{SM}}+4.0177{h_M}}}} \right)} \right] - 0.14511$$

According to the fitting of damage factor in the figure, it can be found that the height of sandy mudstone and mudstone has an effect on the relationship between damage factor and stress level, but it always shows an exponential relationship, i.e., the damage factor increases with the increase of stress level. The degree of influence of damage factor with the change of plastic strain is related to the height ratio of sandy mudstone and mudstone, and the larger the height ratio, the lower the sensitivity to the change of damage factor, which means that the larger the height of sandy mudstone, the smaller the damage of the combination when subjected to the same plastic strain in SM-FL-M.

Before the rock axial stress reaches the peak, the damage occurs inside the rock and the cohesion has been changed, and the initial cohesion is corrected to ${c_{icr}}={c_i}\left( {1 - {D_{cr}}} \right)$. Combining improvement of cohesion and friction angle^31^, and then considering the pre-peak internal damage factor ${D_{cr}}$, the cohesion is weakened to $c=({c_{icr}} - {c_r}){e^{ - {{(\frac{{{{\overline {\varepsilon } }^p}}}{{\varepsilon _{c}^{p}}})}^2}}}+{c_r}$,where ${\overline {\varepsilon } ^p}$is the equivalent plastic strain.

**Fig. 10:**
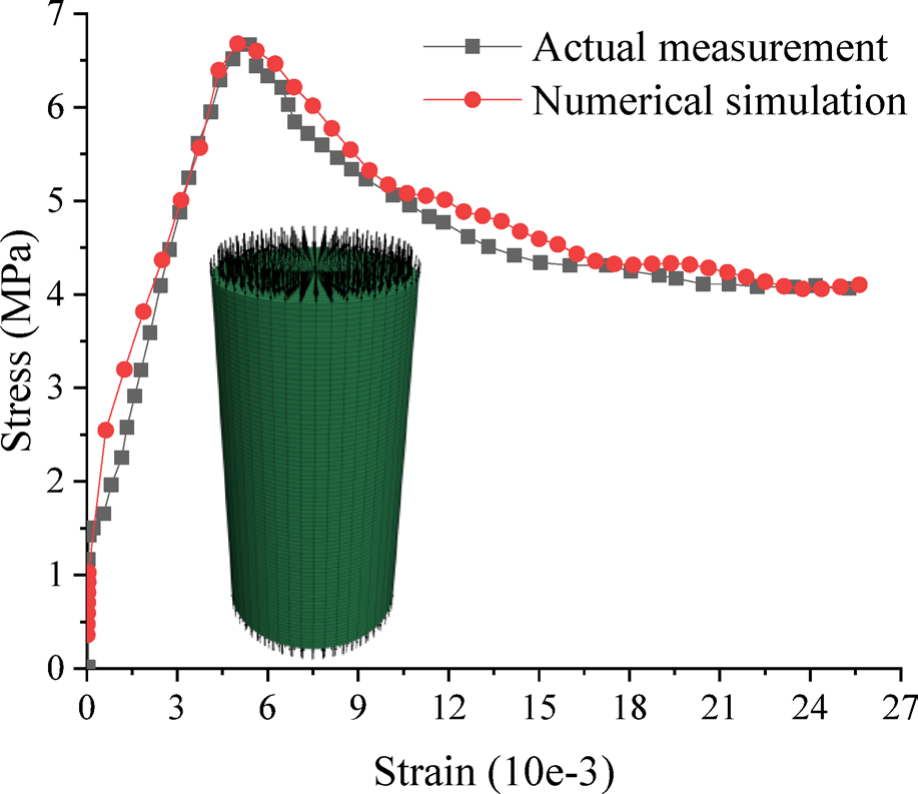
Comparison of uniaxial compression measurements and numerical simulations of standard specimens in filling bodies.

This relationship was brought into the CWFS model for correction, and uniaxial compression numerical simulation was carried out on standard specimens of the filling body, which had the same dimensions as the standard specimens in the laboratory, with a diameter of 50 mm and a height of 100 mm, and the comparison with the actual measurements, as show in Fig. 10. It can be found that both the modulus of elasticity, the peak stress and the corresponding strain, and the residual strength are relatively close to each other, which can simulate uniaxial compression well.

**Fig. 11:**
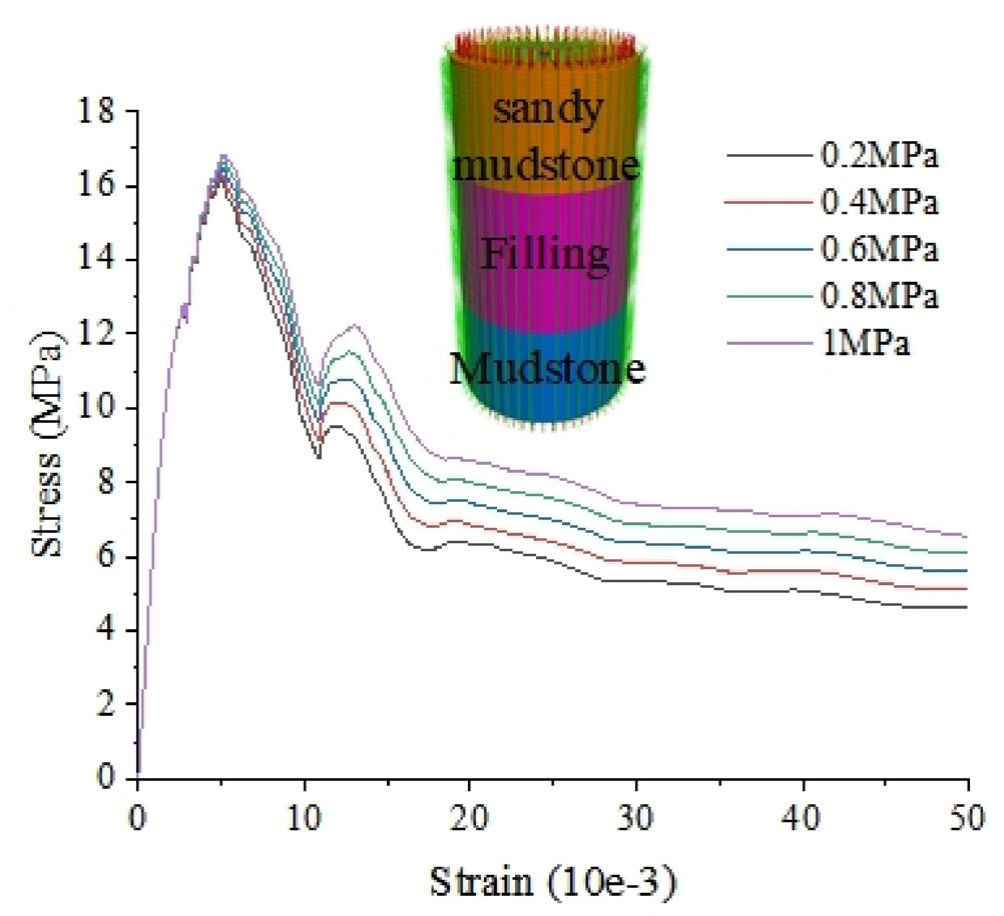
Comparison of uniaxial compression of “SM-FL-M” under different confining pressures.

The stress-strain curves of the assemblage “SM-FL-M” under different confining pressures at the height ratio of 3:4:3 are shown in Fig. 11. It can be found that, when the assemblage is at low confining pressure (0.2, 0.4, 0.6, 0.8, 1 MPa), the higher the confining pressure, the higher the peak stress, the higher the residual strength, and the higher the difference in residual strength. And the phenomenon of sub-peak appears in the simulation, which is due to the lower strength of the filling body, which reaches the residual strength faster after the destruction of the filling body occurs, while the mudstone and sandy mudstone are not completely damaged.

## Conclusions

In this paper, uniaxial loading and cyclic loading/unloading tests are conducted on various combination supports formed by the filling body and surrounding rock in excavation technology without coal pillars in the original roadway filling. The damage characteristics and strength parameters are analyzed, examining the deformation and damage of the combination and its influencing laws from an energy perspective. Additionally, the damage variables of the combination of the three lithologies are discussed. The major conclusions are as follows:

1. The uniaxial compressive strength of SM-FL is negatively correlated with the height ratio of the filler body, and the degree of strength enhancement of the filling increases as the height ratio decreases. In uniaxial compression, the strength of sandy mudstone/mudstone weakens with the increase in its height ratio when the combination of sandy mudstone/mudstone and filling is compressed. The higher the strength of the material combined with the filler, the greater the weakening.
2. When the three lithologic combinations were subjected to uniaxial compression, the strengthening strength of the filling body in SM-FL-M was positively correlated with the height ratio of sandy mudstone and mudstone. In both SM-FL-M and SM-FL + C-M, the degree of weakening of sandy mudstone and mudstone is negatively correlated with the height ratio of sandy mudstone and mudstone.
3. The elastic energy of the different lithologic combinations are all linearly related to the number of cycles, but the fill in FL-M has a faster elastic energy storage rate and the fill has a higher upper storage energy limit than the mudstone. For the same number of cycles, the larger the height difference between sandy mudstone and mudstone in SM-FL-M, the faster the elastic energy storage rate and the higher the upper storage limit. Due to the characteristics of coal, the four lithology combinations have higher dissipation energy for the same number of cycles compared to the three lithology combinations.
4. The heights of sandy mudstone and mudstone in SM-FL-M were analyzed to show an exponential relationship with the damage factor, and the calculation formula was derived, and the greater the height of sandy mudstone, the less the damage of the combination when subjected to the same plastic strain. This relationship was modified to the intrinsic model and verified to accurately simulate the deformation damage of the combined body.

## Data Availability

The datasets used and analysed during the current study available from the corresponding author on reasonable request.
